# Prevalence and consumption pattern of kolanut among pregnant women in Ibadan metropolis

**DOI:** 10.1038/s41598-023-41754-6

**Published:** 2023-09-02

**Authors:** F. A. Atiba, O. A. Popoola, A. A. Odukogbe, A. O. Ihunwo

**Affiliations:** 1 Department of Anatomy, College of Medicine, University of Ibadan, Ibadan, Nigeria; 2 School of Anatomical Sciences, University of the Witwatersrand, Johannesburg, South Africa; 3 Departrment of Community Medicine, University of Ibadan/University College Hospital, Ibadan, Nigeria; 4 Department of Obstetrics and Gynecology College of Medicine, University of Ibadan/University College Hospital, Ibadan, Nigeria

**Keywords:** Health care, Medical research

## Abstract

Kolanut contains caffeine and it is widely consumed in various social contexts in Nigeria and other Sub-Saharan African countries. While some studies have suggested that kolanut is consumed by pregnant women, there is a dearth of information on the prevalence, consumption pattern and reasons for kolanut consumption among this group. This study investigated kolanut use among pregnant women in Ibadan, Oyo State, Nigeria. A cross-sectional study involving 478 consenting pregnant women in all trimesters of pregnancy was conducted. Semi-structured questionnaires were used to collect data. Associations between kolanut use and respondent characteristics were investigated using the chi-square test and logistic regression analysis. The mean age of the women was 28.7 ± 6.3 years. One hundred and sixty-two (33.9%) of women reported kolanut use during pregnancy, 140 (29.3%) in the current pregnancy. Fifty-five (39.3%) pregnant women reported frequent use and 46 (32.9%) used it in high quantities. Significant associations were found between current kolanut use and Hausa respondents (*p* = 0.014), educational level; secondary (*p* = 0.032), tertiary (*p* = 0.006), TBA (*p* = 0.005). The majority (93.7%) used kolanut to prevent spitting, nausea, and vomiting. This study showed that kolanut use is quite common among pregnant women and frequently used in large quantities.

## Introduction

The use of herbal plants, as part of complementary, alternate or traditional medicines during pregnancy has been on the increase^1–3^ regardless of adequate scientific evidence about the safety of these substances^4^ can cause harmful effects on the developing fetus^5,6^. The reported use of herbs and alternate medications, during pregnancy by pregnant mothers to suppress symptoms of morning sickness is on the increase, in various communities^1,2,4,7^. These herbal substances/ medications are believed by many to be cheaper than conventional medications, natural and safe, reasons that explain their increased use^8^. In addition to easy accessibility at very low cost and the assumption that they are safe, the proclamation by the WHO in 2019^9^ encouraging traditional/herbal and/or alternate medications may have made their use more attractive. About 80.0% of the world population takes alternate medicines to treat a disease or alleviate the response of a disease^10^.

More than half of pregnant women experience morning sickness during pregnancy and this manifests as tiredness, dizziness, nausea and vomiting^11,12^. Although termed morning sickness, this group of symptoms can occur at any time of the day, depending on individual hormonal responses. Majority of pregnant women take different herbs, traditional and/or alternates substances^13,14^, to alleviate these symptoms which are more common in the first trimester. These substances can be in form of drugs prescribed by physicians or pharmacists, or self-prescription^15^, resulting from their observation of other pregnant women or practices passed down by their mothers or relatives^4^. The prevalence of consumption of herbal and alternate medicines by pregnant women is between 12.0% and 90.0% during pregnancy^8,14,16^. These alternate substances, which could be in the form of herbs or traditional plants^2,16,17^, include kolanut^17,18^. Kolanut, a plant and very common social snack, consumed by every gender and age group throughout tropical and equatorial Africa including Nigeria, is equivalent to tea, coffee, maté and cocoa^19–21^. Kolanut has 4 main different species and the most common one to the Southwest of Nigeria is the Gbanja which comes in two colors, white and red^19,22^. Kolanut is dark brown in color when bitten, acidic taste when fresh and bitter when dry, with an increase in nutmeg and aromatic aroma^23^. Kolanut contains different fractions like catechins, procyanidins, sugar, sterols, fatty acids, alkaloids, kolanin, theobromine and caffeine in large quantity^24^. Kolanut has numerous uses, apart from being a social snack, it has been documented to reduce labor pain, swellings, and accelerate the healing of fresh wounds^25^. Because kolanut contains caffeine, it is considered as a strong Central Nervous System Stimulant and increases sympathetic nervous system^26,27^. Caffeine overuse in pregnancy can cause death through ventricular fibrillation and cardiac arrest^21^. It also reduces the estrogen level in the female thereby affecting conception, gestational period and delivery, as a result affecting fetal growth leading to stunting.^28–30^ Kolanut also cause toxic effects such as delay in neuronal migration in the developing brain^8,31^. It has also been established that the effects of these uncensored substances are dose dependent, and the time of consumption could magnify its effects^32^. However, these results were not found in humans but could be potentially the same in humans.

Therefore, this research was designed to document the prevalence and consumption pattern of kolanut (onset of use, amount consumed, duration of use and trimester of use), common reasons for its use during pregnancy, the perceived and reported effects in pregnancy, factors associated with its use in pregnancy and its local geographical distribution.

## Methods

### Study area

The study was conducted in Ibadan, the most populous city in Oyo State, Nigeria with over 3 million people (National Population Commission, 2016). Ibadan is the third most populous city in Nigeria after Lagos and Kano, and the country's largest city by geographical area. The city has 11 local government areas (LGAs); five of these (Ibadan North, Ibadan North-East, Ibadan North -West, Ibadan South- East, Ibadan South -West) constitute the core Ibadan municipality and the other six (Akinyele, Egbeda, Ido, Lagelu, Ona-Ara and Oluyole) are the outlying areas which make up the rest of Ibadan city.

### Study population

Participants in this study were women attending antenatal care at formal or informal health care facilities in the study area. These women had pregnancies confirmed by an obstetric ultrasound scan. We excluded women who were unable to communicate in any of the common local languages and children under the age of 15 years.

### Sampling technique

Two LGAs were purposively selected for this study; Ibadan North due to its urban nature and mix of tribes (Ibo, Hausa, and Yoruba) and Akinyele as the peri urban LGA. A list of health care providers/facilities was obtained from the State Ministry of Health. One tertiary hospital, one secondary hospital, two primary health care centres and two TBA centres were selected in each LGA using simple random sampling techniques. The sample size was proportionally allocated across the selected facilities based on the number of registered antenatal attendees in the facility during the preceding year. At facility level, informed consent was obtained from adult respondents and guardians for individuals less than 21 years of age. Consenting women were recruited consecutively until the facility sample size was achieved.

### Sample size

The sample size for this study was determined to estimate a proportion using a prevalence of 10.0% with a two-sided alpha of 5.0%, a difference of 4.0% and a non-response rate of 20.0%. This yielded a minimum sample size of 271 respondents. We approached 500 women, and 22 questionnaires were unsuited to analysis due to grossly incomplete responses and these were excluded from analysis leaving us with 478 respondents.

### Data collection

This cross-sectional study utilised a structured questionnaire developed by the researchers. The content validity was ensured through extensive review of relevant literature on previous research reports to develop the relevant questions. The questionnaire was pre-tested with 30 pregnant women in two public and TBA facilities outside the selected LGAs. Following the pre-test, appropriate modifications were made to the instrument, ambiguous terms modified, and unnecessary items deleted. The questionnaire contained sixteen (16) questions on socio demographic characteristics, obstetric history, history of index pregnancy and pattern of kolanut consumption; with the frequency of use defined as high (every day), medium (thrice a week), low (≤ once a week) and quantity of use defined as high (≥ 1 nut at once), medium (half to < 1 lobe) and low (quarter to < half lobe). The questionnaire was interpreted and administered to the participants by four trained female research assistants. The assistants were trained to conduct initial entry/engagement on REDCap®, obtain informed consent and information on the required variables from interaction with respondents. The quality of information collected was cross-checked for any error and problems discovered were resolved by the supervisor on the field. All the questionnaires administered during the study were given code numbers and no names were recorded. The data collected from the pregnant women performed in accordance with relevant guidelines and regulations based on code of conducts from the ethical committee. Collection of data was carried out within a period of four months.

### Mapping of geographical kolanut consumption

This was done by extracting the coordinates from Google mapping of all the addresses of the respondents and these coordinates were entered into a Microsoft excel and saved as Comma Separated Values (CSV) alongside other variables and was exported into ArcGIS online to generate the maps. Also, the hotspots were generated based on the number of pregnant women who consumed kolanut, while each clustered color represents the rate of kolanut consumption in that Local Government.

### Statistical analysis

Data were recorded electronically on the REDCap® platform using android tablets, cleaned, and analysed with the STATA package. Continuous variables were summarised as means with standard deviations or medians with interquartile ranges. Categorical variables were summarised as proportions. Chi square tests were used to investigate associations between kolanut use and categorical variables. Univariate and multiple logistic regression was fitted to identify independent factors associated with kolanut use, and odds ratios and 95% confidence intervals were reported. The level of significance for all tests was at < 5%.

### Ethical approval

Ethical approval for this study was obtained from the University of Ibadan/University College Hospital, Ibadan Ethical Review Committee and permission from the Ministry of Health, Oyo State, Nigeria. Also, consent to participate was obtained from the participants. All the principles of ethical conduct of research were strictly adhered to.

### Consent for publications

This was obtained from the participants and authors.

## Results

### Sociodemographic characteristics

There were 478 pregnant women with a mean age of 28.7 ± 6.3 years. Most of the respondent were Yoruba, 82.0% (392), while 9.2% (44) were Hausa, 5.7% (27) Igbo and others constituted 3.1% (15). More than half, 50.4% (241) of the study population were adherents of Islam, 49.2% (235) were Christians and 0.4% (2) had other faiths. The largest educational group were women with secondary level education 50.8% (243), while 43.9% (210) were semi-skilled workers (Table 1).

**Table 1:** Distribution of sociodemographic characteristics (n = 478).

Variable	Frequency	%
Age (in completed years)		
15–24	124	25.9
25–29	136	28.5
30–34	129	27.0
35–45	89	18.6
Ethnicity		
Yoruba	392	82.0
Ibo	27	5.7
Hausa	44	9.2
Others	15	3.1
Religion		
Christianity	235	49.2
Islam	241	50.4
Others	2	0.4
Occupation		
None	25	5.2
Unskilled	46	9.6
Semiskilled	210	43.9
Skilled	128	26.8
Professional	69	14.4
Educational level		
None (No formal education)	21	4.4
Primary School	34	7.1
Secondary School (or equivalent completed)	243	50.8
Tertiary (or equivalent completed)	180	37.7

### Obstetric history

Out of the pregnant women, 11.1% (53) were in their first trimester, 34.7% (166) in their second semester and 54.2% (259) in their third trimester. Also, 66.5% (318) were multiparous (≥ 2), 28.2% (135) were primiparous and 5.2% (25) nulliparous (Table 2).

**Table 2:** Obstetrics history of respondents (n = 478).

Facility type	Frequency	%
Tertiary	108	22.6
Secondary	86	18.0
Primary	185	38.7
TBA/Faith Clinic	99	20.7
Trimester		
1^st^	53	11.1
2^nd^	166	34.7
3^rd^	259	54.2
Parity		
Nulliparous	25	5.3
Primiparous	135	28.2
Multiparous (≥ 2)	318	66.5
Number of Miscarriages		
0	406	84.9
1	69	14.4
≥ 2	3	0.6
Number of Children		
0–1	157	32.8
2–3	205	42.9
4–8	104	21.8
Missing	12	2.5

### Prevalence of kolanut consumption

The study showed that 33.9% (55) respondents had ever used kolanut at different pregnancies while 29.3% (41) respondents were currently using kolanut. Out of the one hundred and sixty-two respondents that ever took kolanut, 40.1% (65) of them took kolanut during the first trimester, 17.9% (29) used in the second trimester while 7.4% (12) used in the third trimester. However, in the present pregnancy, 84.3% (118) took kolanut in the first trimester, 8.6% (12) had in second trimester and the least, 0.7% (1) pregnant woman in the third trimester and missing 0.7% (1).

### Pattern of kolanut consumption

The frequency of consumption of kolanut in the present pregnancy (n = 140) was, 39.3% (55) of the pregnant women consumed kolanut every day, 46.4% (65) consumed thrice a week, while 6.4% (9) consumed once a week and 7.9% (11) did not provide information on consumption frequency. The quantity of kolanut used in this present pregnancy showed that 32.9% (46) consumed up to one nut at once, 45.0% (63) consumed more than half but less than one lobe, while 11.4% (16) of the respondents consumed quarter to half lobe while 10.7% (15) were missing. For the ever use pregnancy (n = 162), 46.9% (76) consumed every day, 38.3% (62) consumed thrice a week while 6.8% (11) consumed once a week and 8.0% (13) were missing. The quantity of use of the ever use kolanut was 42.0% (68) consumed up to one nut at once, 38.9% (63) consumed more than half but less than one lobe while 10.5% (17) consumed quarter to half lobe and 8.6% (14) were missing (Table 3).

**Table 3:** Frequency and quantity of kolanut use in current (n = 140) and ever pregnancy (n = 162).

Frequency of use	Frequency Current Use (n = 140)	%	Frequency Ever Use (n = 162)	%
High	55	39.3	76	46.9
Average	65	46.4	62	38.3
Low	9	6.4	11	6.8
Missing	11	7.9	13	8.0
Total	140	100.0	162	100.0
Quantity of use				
High	46	32.9	68	42.0
Average	63	45.0	63	38.9
Low	16	11.4	17	10.5
Missing	15	10.7	14	8.6
Total	140	100.0	162	100.0

### Reasons for kolanut use

Over half, 56.8% (92) of the pregnant women used kolanut due to excessive salivation, 37.0% (60) due to nausea or vomiting, 3.1% (5) of women used it for cough and for recreation. A small number, 2.5% (4) of them used it to stop dizziness, 1.2% (2) used it for no reason, 0.6% (1) used it to ‘prepare’ the womb for easy delivery, while very few (0.6%) (1) used it because they sell the kolanut.

### Benefits and perceived usefulness of kolanut

Most women, 90.0% (146) reported that kolanut consumption helped in reducing excessive salivation, 71.6% (116) for nausea and 16.1% (26) for prevention of vomiting. 3.7% (6) of the women claimed it helped in reducing cough, 2.5% (4) reported it refreshes their mouth while 3.6% (6) did not provide any benefit.

### Sources of introduction to kolanut use in current pregnancy

Kolanut was introduced to the pregnant women, 40.7% (57) by friends, 37.0% (52) by relatives, 13.0% (18) decided to use it of their own volition and 1.8% (3) by a doctor or nurse while none heard from the media and 7.5% (10) did not provide an answer.

### Association between kolanut use and variables

Table 4 combines results of a crude and adjusted odds of kolanut use various sociodemographic variables and facility of recruitment of study participants. Odds are adjusted for age group, tribe, religion, employment, education experience, trimester at interview, parity and facility of recruitment. Crude odds show Ibo women were significantly less likely to eat kolanut in current pregnancy compared to Yoruba women (OR = 0.16, 95% CI = 0.04–0.70, *p* = 0.015) in the analysis but after adjustment, Hausa women were now significantly less likely than Yoruba women to report kolanut use (AOR = 0.31, 95% CI = 0.12–0.79, *p* = 0.014). Higher levels of education were associated with reduced risk of kolanut use. Women with secondary or tertiary education were significantly less likely to report kolanut use (AOR = 0.32, 95% CI = 0.11–0.91 and AOR = 0.21, 95% CI = 0.07–0.63 respectively) compared to women with no formal education. Respondents recruited from TBAs, or faith clinics had significantly higher odds of kolanut use compared to women who attended tertiary health facilities AOR = 3.17, 95% CI = 1.41–7.09.

**Table 4:** Bivariate and Adjusted Odds of factors associated with Kolanut use in current pregnancy – bivariate and adjusted odds.

Variable	Bivariate			Multivariate		
	Odds ratio (OR)	95% CI	P value	Odds ratio (AOR)	95% CI	P value
Age						
15–24	1			1		
25–29	1.38	0.80–2.36	0.247	1.57	0.81–3.03	0.180
30–34	1.54	0.90–2.65	0.118	1.69	0.80–3.60	0.176
35–45	0.78	0.41–1.49	0.453	0.78	0.31–1.97	0.597
Ethnicity						
Yoruba	1			1		
Ibo	0.16	0.04–0.70	0.015	0.31	0.07–1.47	0.139
Hausa	0.45	0.21–1.00	0.051	0.31	0.12–0.79	0.014
Others	0.15	0.02–1.12	0.064	0.16	0.02–1.44	0.103
Religion						
Christianity	1			1		
Islam	1.37	0.92–2.03	0.125	1.03	0.64–1.63	0.916
Work experience						
None	1.19	0.42–3.33	0.741	0.79	0.23–2.70	0.704
Unskilled	1.21	0.52–2.80	0.665	0.36	0.12–1.04	0.060
Semiskilled	1.14	0.61–2.13	0.683	0.54	0.25–1.17	0.120
Skilled	1.72	0.89–3.31	0.107	0.69	0.30–1.59	0.381
Professional	1			1		
Educational level						
None (No formal education)	1			1		
Primary School	0.38	0.12–1.17	0.092	0.32	0.08–1.20	0.091
Secondary School (or equivalent completed)	0.45	0.19–1.12	0.085	0.32	0.11–0.91	0.032
Tertiary (or equivalent completed)	0.24	0.10–0.62	0.003	0.21	0.07–0.63	0.006
Facility type						
Tertiary	1			1		
Secondary	1.17	0.57–2.37	0.674	0.85	0.37–1.91	0.687
Primary	1.81	1.02–3.24	0.044	1.63	0.77–3.44	0.202
TBA/Faith Clinic	4.14	2.22–7.74	< 0.001	3.17	1.41–7.09	0.005
Trimester distribution						
1^st^	1			1		
2^nd^	0.69	0.35–1.37	0.291	0.76	0.36–1.61	0.475
3^rd^	0.98	0.52–1.85	0.953	0.93	0.45–1.91	0.849
Parity						
Primiparous	1			6.78	0.14–323.31	0.332
Multiparous	1.17	0.75–1.83	0.486	1		
Nulliparous	0.22	0.05–0.99	0.048	2.01	0.04–96.77	0.724

### Distribution of kolanut consumption

**Figure 1:**
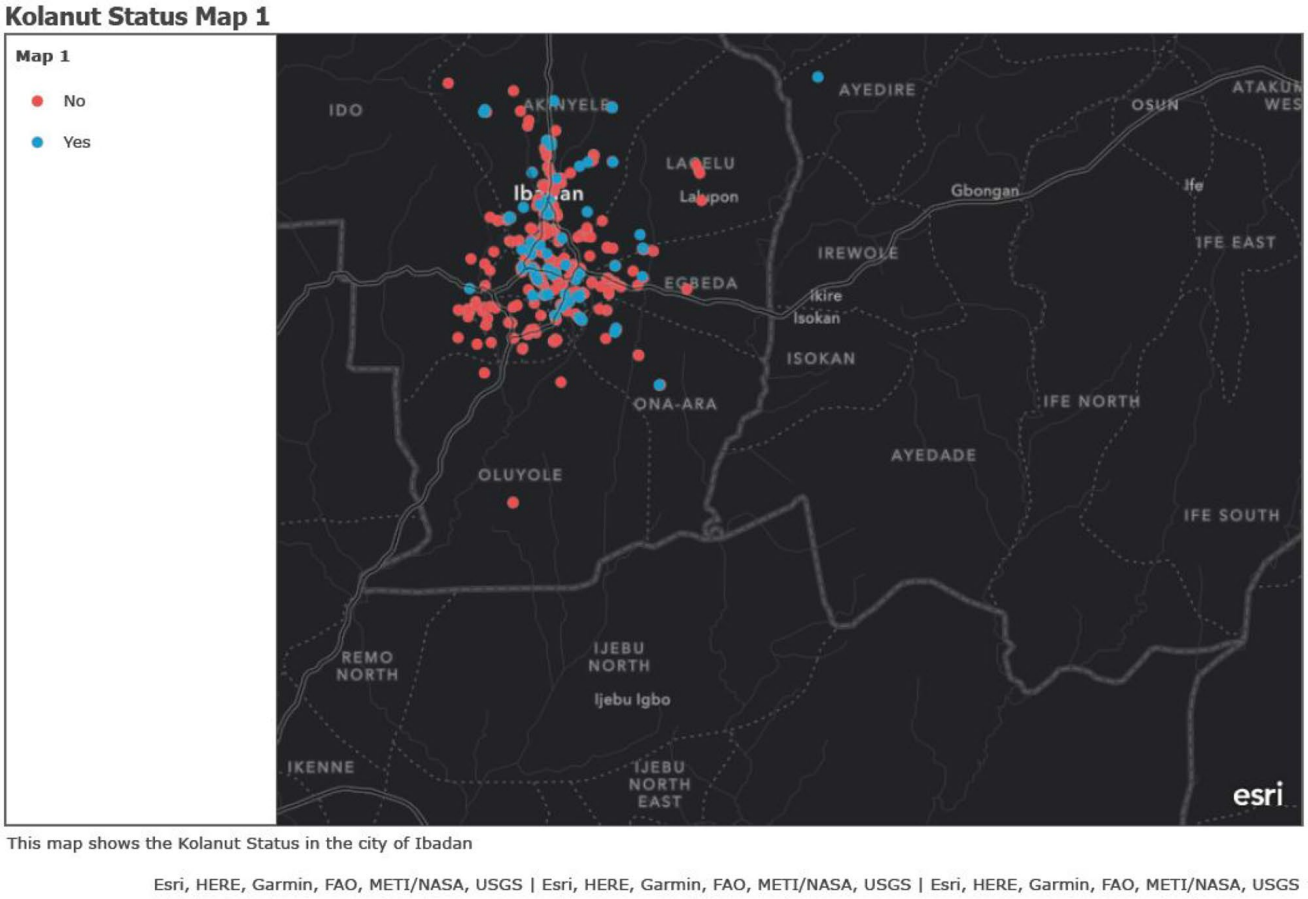
Distribution of kolanut consumption within respondents’ residential Local governments. https://arcg.is/1XukTW0.

**Figure 2:**
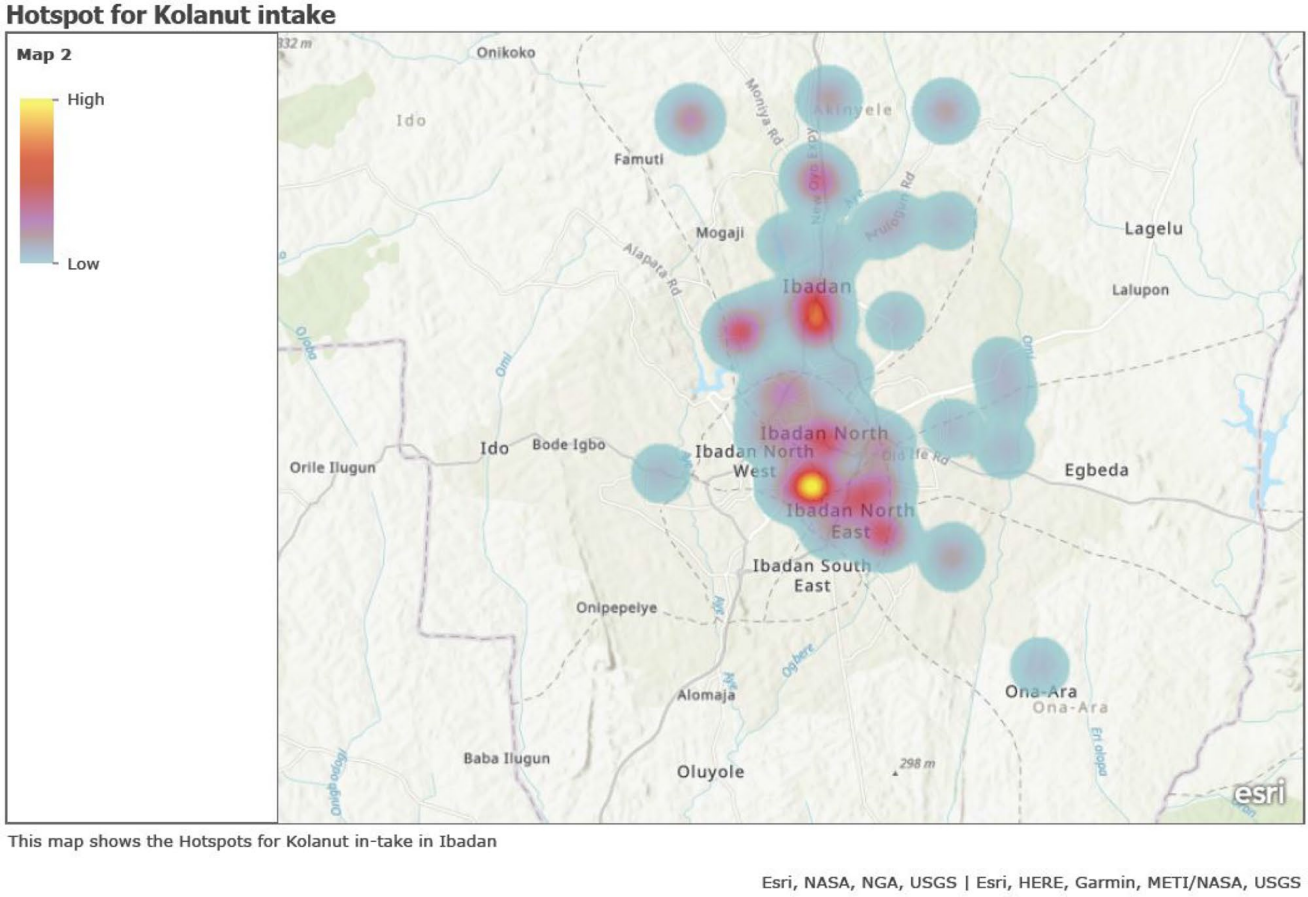
Hotspots distribution of kolanut consumption within respondents’ residential Local governments. https://arcg.is/r4ufa0.

The distribution of kolanut consumption was shown in Fig. 1 and this signifies the respondents’ residential addresses. The blue dots indicated the location of those that consumed kolanut in pregnancy and red dots showed those that did not consume kolanut in pregnancy within their residential Local Government areas. Higher percentage of kolanut consumption was more in the pregnant women as depicted as hotspots in Ibadan Northeast and Ibadan North Local Government Areas and followed by Akinyele Local Government area, while there is a low consumption rate at Ona Ara and Egbeda Local Governments areas based on the respondents’ residential addresses (Fig. 2).

The TBAs/Faith clinic and the primary health facilities had higher number of kolanut use attending pregnant women than other facilities.

## Discussion

This study confirms that kolanut is frequently consumed at relatively high quantity by pregnant women. The current study showed a prevalence of 33.9% of kolanut consumption in previous pregnancy and 29.3% reported use in the current pregnancy, this shows a high consumption of kolanut in pregnancy. This is similar to the report from the study conducted in Jos, North Central Nigeria^33^, which reported a prevalence of 61.9%. Also, Ifesanya and Oke^18^ reported a prevalence of 44.2% of kolanut/bitter kola consumption in pregnancy while looking at adverse gingival conditions among pregnant women in South-West Nigeria. However, other Nigerian studies that collected kolanut consumption data among pregnant women reported lower prevalence of kolanut use, this could be because kolanut consumption in pregnancy was not their primary focus or their geographical location and culture do not encourage kolanut consumption. Fawale and his colleagues^34^, investigated Restless Legs Syndrome in Hausa/Fulani women in Northern Nigeria and reported a prevalence of 8.4%. Also, a report on self-medication among women in South-South Nigeria found that only 1.0% of women reported kolanut intake in pregnancy^35^In Ghana, a neighboring West African country, Abu et., al^36^, in a study of pica use in pregnancy found that 3.6% of women reported kolanut use. The present study showed a higher rate of kolanut use compared to those studies in Northern and South-South Nigeria probably because our focus on this study was on kolanut consumption pattern unlike other studies who had other primary focuses.

Yoruba pregnant women had a higher percentage of kolanut consumption than another ethnic group. The reasons could include the availability of kolanut in some regions compared to others and cultural beliefs that could promote use in pregnancy. However, the ethnic differences found in this study must be interpreted with caution given the inadequate sample sizes for non-Yoruba ethnic groups.

The most reported reasons for use were for the prevention of excessive salivation, nausea, and vomiting, reported in about 56.0% and 37.0% respectively of women that reported ever using kolanut. And it has been established by different studies that these symptoms constitute the term morning sickness which usually occur in the first trimester but not limited to this period^11,12,37^ and due to this, some pregnant women seek medications and/or herbs to mitigate these symptoms. This is why these women sought after kolanut especially because of its bitter taste which helps to stop the excessive salivation, nausea and vomiting. In support of the importance of nausea and vomiting warranting kolanut use, a study in North Central Nigeria reported that preventing nausea and vomiting was the reason mentioned by all women reporting kolanut use in pregnancy^33^.

Moreover, in other studies outside Nigeria focusing on caffeine use and not kolanut specifically reported that almost all women take caffeine containing foods for different reasons at different points of pregnancy^30,38,39^. For example, an Ethiopian study^39^ investigated the consumption of caffeinated beverages and foods in a community sample of pregnant women and found that 98.2% of the beverages and foods had caffeine; with 17.6% of the women reporting excessive caffeine intake. These studies of caffeine use that are not restricted to kolanut alone highlight the possibility of greater caffeine consumption in pregnancy due to other sources such as coffee, energy drinks, soda such as Coca cola, and tea.

However, a significant number of studies have reported higher risk of adverse pregnancy outcomes such as miscarriages^28,38,40–42^, among pregnant women consuming high levels of caffeine-containing substances in pregnancy like kolanut. Caffeine can cross the placenta into the amniotic fluid and the fetus, resulting in adverse pregnancy outcomes^43^, such as small for gestational age birth^29^, a delay in neuronal migration by retention of external granular cells in the cerebellum and neuronal toxicity which can affect posture, balance and vision^31^, and these effects can be elicited by kolanut due to its high content of caffeine.

Kolanut use in this study was most frequently reported in the first trimester (32.1%). Most women studied reported medium (thrice in a week) or high frequency (daily) kolanut use, while most women also reported high (at least 1 nut at once) or medium (half to less than 1 lobe) quantity. These findings suggest that users tend to consume significant quantities at relatively frequently and highlight the need for efforts to identify these women and offer interventions that will get them to discontinue use. Health providers have a crucial role to play in this regard, especially given that history of kolanut use may not be routinely asked during antenatal care, a concern similarly shared by other authors^44,45^. Additionally, women who are managed for nausea and vomiting, have a greater chance of using kolanut for treatment and could be important targets for identifying those who may be using kolanut. Hämeen-Anttila et.al.,^46^ showed that the need for health information was significantly higher among pregnant women using herbal preparations, supporting the argument for the provision of health information to women who may be using substances like kolanut. However, this present study showed that 1.8% of pregnant women were advised by medical practitioners to consume kolanut to alleviate the symptoms of morning sickness. This act must stop and be discouraged.

Our study also noted that kolanut use was significantly higher among women with lower educational level, women attending primary health care centers and TBA clinics, after adjusting for other demographic characteristics and reproductive health related variables. The higher rates among women with lower education could be due to poor knowledge of foods safe for consumption in pregnancy^47^, compared to more educated women. It could also reflect poor socioeconomic status of women with lower education who may lack funds to seek healthcare for treatment of symptoms such as nausea and vomiting, thereby, visiting PHCs and TBA clinics, resulting in higher amount of kolanut use in these centers. In addition to women with excessive salivation and nausea and vomiting, interventions aimed at reducing kolanut use in pregnancy need to target women in PHCs or TBA clinics, Yoruba women, and those with lower education.

An understanding of patterns of kolanut use and associated factors as provided from our findings will support the design of interventions that could target pregnant women with kolanut, or caffein-containing foods use in pregnancy in Nigeria. Significant associated factors like ethnicity, with the highest use among Yoruba women, those with lower educational levels and those attending antenatal care at primary health care centers or TBAs especially within Ibadan Northeast and Ibadan North Local Government areas are to be included.

It is worthy of note to state that there is a limited number of studies on kolanut consumption in pregnancy, several studies have examined psychoactive substances use among secondary school students with lifetime use as high as 86.0%^48^ and 63.5%^49^ in South-West and South-East Nigeria, respectively. Another study of students however reported 18.8% from the South-East^50^. Altogether, these studies of kolanut use in diverse populations suggest that kolanut use is popular in Nigeria and as stated by previous authors, some of the reasons for this include that it is cheap, readily available, and socially acceptable^33,48^.

### Limitations of the study

The limitations of this study include the potential for recall bias concerning kolanut use in previous pregnancies. Additionally, some of the women studied in the first and second trimester could use kolanut later in their pregnancy, potentially underestimating kolanut use. However, majority of the women studied were in the third trimester and most current kolanut use would have been captured. In addition, the assessment of ever-use of kolanut is likely to capture most women that use the substance. Other limitations include the inability to quantitatively estimate the amount of the kolanut use as there are currently no guidelines for determining levels that are likely to result in adverse outcomes. This contrasts with caffeine-containing foods in the developed world such as coffee and soda that have been extensively studied.

## Conclusion

This study has shown that kolanut use is quite common among pregnant women and frequently used in large quantities. Interventions need to focus on Yoruba women, those with nausea and vomiting, less educated women, and those attending lower levels of healthcare with education on the possible adverse effects of kolanut consumption in pregnancy. The results of this study justify research into consequences of kolanut use in pregnant women.

## Recommendations

Studies designed with large sample sizes of the ethnic groups and including qualitative methodology are needed to understand kolanut use patterns in pregnancy by ethnicity. Also, multiple sources of caffeine in the Nigerian context and estimation of total caffeine intake through kolanut should be studied. Furthermore, health information about hazards of substances like kolanut or foods containing caffein should be included in routine counseling in ANC clinics and public awareness inclusive. Finally, further studies with a wider coverage within the country should be considered as this would give a better picture of kolanut consumption among pregnant women.

## Data Availability

The datasets used and/or analyzed during the current study are available from the corresponding author on reasonable request.

## Abbreviations

LGA: Local government area
PHC: Primary health centre
TBA: Traditional birth assistant
WHO: World health organization
CSV: Comma separated values

## References

[1] El Hajj M, Holst L (2020). Herbal medicine use during pregnancy: A review of the literature with a special focus on Sub-Saharan Africa. Front. Pharmacol..

[2] Onyiapat JLE, Okoronkwo IL, Ogbonnaya NP (2011). Complementary and alternative medicine use among adults in Enugu. Nigeria. BMC Complement. Altern. Med..

[3] Kennedy DA, Lupattelli A, Koren G, Nordeng H (2016). Safety classification of herbal medicines used in pregnancy in a multinational study. BMC Complement. Altern. Med..

[4] Mohammed MA, Ahmed JH, Bushra AW, Aljadhey SH (2013). Medications use among pregnant women in Ethiopia: A cross sectional study. J. Appl. Pharm. Sci..

[5] Chung BHY (2011). Teratology and developmental pharmacology: Why should paediatricians care?. Hong Kong J. Paediatr..

[6] Atiba FA, Imosemi IO, Malomo AO (2017). Noxious effect of Moringa oleifera leave extract on the developing brain, morphology and behaviour of Wistar rat. Ital. J. Anat. Embryol..

[7] Kennedy DA, Lupattelli A, Koren G, Nordeng H (2013). Herbal medicine use in pregnancy: Results of a multinational study. BMC Complement. Altern. Med..

[8] Yildirim M, Desdicioglu R, Kara H, Avsar AFY (2016). Gebelikte Bitkisel Ürünlerin Kullanımı. Ankara Med. J..

[9] World Health Organization. *WHO global report on traditional and complementary medicine*. (2019).

[10] Peltzer K, Pengpid S (2018). Prevalence and determinants of traditional, complementary and alternative medicine provider use among adults from 32 countries. Chin. J. Integr. Med..

[11] Wodi C, Danborno B, Sunday AS, Eze UA (2014). Incidence of nausea and vomiting of pregnancy among Nigerian women. Sch. J. Appl. Med. Sci..

[12] Ugoma DE (2018). Incidence of pre-pregnancy and pregnancy-related illnesses in rural women accessing antenatal care services in Awka, south-east. Nigeria. J. Public Heal. Epidemiol..

[13] Ebrahimi N, Maltepe C, Einarson A (2010). Optimal management of nausea and vomiting of pregnancy. Int. J. Womens. Health.

[14] Mawoza T, Nhachi C, Magwali T (2019). Prevalence of traditional medicine use during pregnancy, at labour and for postpartum care in a rural area in Zimbabwe. Clin. Mother Child Heal..

[15] Yusuff KB, Omarusehe LD (2011). Determinants of self medication practices among pregnant women in Ibadan. Nigeria. Int. J. Clin. Pharm..

[16] Mudonhi N, Nunu WN (2022). Traditional medicine utilisation among pregnant women in Sub-saharan African Countries: A systematic review of literature. INQUIRY.

[17] Ologe M (2008). Herbal use among pregnant mothers in Ilorin, Kwara State. Nigeria. J. Obstet. Gynaecol. (Lahore).

[18] Ifesanya JU, Oke GA (2013). Self report of adverse gingival conditions among pregnant South-Western Nigerian women. J. Dent. Oral Hyg..

[19] Tachie-Obeng, E. & Brown, N. Kolanuts (Cola nitida & Cola acuminata). in *The key non-timber forest products of Central Africa: state of the knowledge.* (eds. Clark, L. & Sunderland, T.) 87–120 (SD Publication Series Office of Sustainable Development Bureau for Africa, US Agency for International Development, 2004).

[20] Burdock. *Kola nut (Kola nut). In: Fenaroli’s Handbook of Flavor Ingredients*. (CRC Press, Boca Raton, 2005).

[21] Starin D (2013). Kola nut: So much more than just a nut. J. R. Soc. Med..

[22] Erinfolami A (2011). Prevalence and associated risk factors of Kola nut chewing among secondary school students in Osogbo. Nigeria. Ment. Illn..

[23] Burdock G, Carabin I, Crincoli C, Al E (2009). Safety assessment of kola nut extract as a food ingredient. Food Chem. Toxicol..

[24] Adesanwo JK, Ogundele SB, Akinpelu DA, McDonald AG (2017). Chemical analyses, antimicrobial and antioxidant activities of extracts from Cola nitida seed. J. Explor. Res. Pharmacol..

[25] Miller R.A. *The Magical and Ritual Use of Herbs*. (Inner Traditions International, Limited, 1979).

[26] Rice D, Barone S (2000). Critical periods of vulnerability for the developing nervous system: Evidence from humans and animal models. Environmental Health Perspectives.

[27] Blumenthal, M. *et al.* The Complete German Commission E Monographs: Therapeutic Guide to Herbal Medicines (CD-ROM). *American Botanical Council: 9780967077222:* (1998). Available at: https://www.amazon.com/Complete-German-Commission-Monographs-Therapeutic/dp/0967077222. (Accessed: 2nd July 2020)

[28] Gaskins AJ (2018). Pre-pregnancy caffeine and caffeinated beverage intake and risk of spontaneous abortion. Eur. J. Nutr..

[29] Modzelewska D (2019). Caffeine exposure during pregnancy, small for gestational age birth and neonatal outcome - Results from the Norwegian mother and child cohort study. BMC Pregnancy Childbirth.

[30] Lamy S, Houivet E, Benichou J, Marret S, Thibaut F (2020). Caffeine use during pregnancy: prevalence of use and newborn consequences in a cohort of French pregnant women. Eur. Arch. Psychiatry Clin. Neurosci..

[31] Atiba FA, Fatokun AA, Imosemi IO, Malomo AO (2021). Kola nut from Cola nitida vent. Schott administered to pregnant rats induces histological alterations in pups’ cerebellum. PLoS One.

[32] Källén B (2016). Drugs during pregnancy: Methodological aspects. Drugs Dur. Pregnancy Methodol. Asp..

[33] Nyango D, Daru P, Mutihir J (2012). Substance abuse among antenatal patients at Jos university teaching hospital, north central Nigeria. J. West Africa Coll. Surg..

[34] Fawale M (2018). Restless legs syndrome: A rarity in the Nigerian pregnant population?. Sleep Med..

[35] Abasiubong F (2012). Self-medication: Potential risks and hazards among pregnant women in Uyo. Nigeria. Pan Afr. Med. J..

[36] Abu BAZ, van den Berg VL, Raubenheimer JE, Louw VJ (2017). Pica practices among apparently healthy women and their young children in Ghana. Physiol. Behav..

[37] Einarson TR, Piwko C, Koren G (2013). Quantifying the global rates of nausea and vomiting of pregnancy: A meta-analysis. J. Popul. Ther. Clin. Pharmacol..

[38] Chen L (2014). Exploring maternal patterns of dietary caffeine consumption before conception and during pregnancy. Matern. Child Health J..

[39] Alamneh AA, Endris BS, Gebreyesus SH (2020). Caffeine, alcohol, khat, and tobacco use during pregnancy in Butajira. South Central Ethiopia. PLoS One.

[40] Chen LW (2016). Maternal caffeine intake during pregnancy and risk of pregnancy loss: A categorical and dose-response meta-analysis of prospective studies. Public Health Nutr..

[41] Hahn KA (2015). Caffeine and caffeinated beverage consumption and risk of spontaneous abortion. Hum. Reprod..

[42] Li J (2015). A meta-analysis of risk of pregnancy loss and caffeine and coffee consumption during pregnancy. Int. J. Gynecol. Obstet..

[43] Baptiste-Roberts K, Leviton A (2020). Caffeine exposure during pregnancy: Is it safe?. Semin. Fetal Neonatal Med..

[44] Illamola SM (2019). Use of herbal medicine by pregnant women: What physicians need to know. Front. Pharmacol..

[45] Fakeye TO, Adisa R, Musa IE (2009). Attitude and use of herbal medicines among pregnant women in Nigeria. BMC Complement. Altern. Med..

[46] Hämeen-Anttila K (2015). Factors associated with the need for information about medicines among pregnant women - A multinational internet-based survey. Res. Soc. Adm. Pharm..

[47] Fasola O, Olayinka A, Fasola F (2018). Knowledge, attitude and practice of good nutrition among women of childbearing age in Somolu Local Government. Lagos State. J. Public Health Africa.

[48] Oshodi OY, Aina OF, Onajole AT (2010). Substance use among secondary school students in an urban setting in Nigeria: Prevalence and associated factors. African J. Psychiatry (South Africa).

[49] Manyike PC (2016). Correlates for psycho-active substance use among boarding secondary school adolescents in Enugu, South East, Nigeria. BMC Pediatr..

[50] Ibeneme C, Nwala Gabriel C, Ojinnaka Ngozi C (2021). Pattern of adolescent substance abuse among secondary school students in Umuahia south eastern Nigeria oJTQ. J. Addict. Behav. Ther. Rehabil..

